# Macrophages inhibit adipogenic differentiation of adipose tissue derived mesenchymal stem/stromal cells by producing pro-inflammatory cytokines

**DOI:** 10.1186/s13578-020-00450-y

**Published:** 2020-07-20

**Authors:** Hui Ma, Ya-nan Li, Lin Song, Rui Liu, Xiaolei Li, Qianwen Shang, Ying Wang, Changshun Shao, Yufang Shi

**Affiliations:** 1 The First Affiliated Hospital of Soochow University, Institutes for Translational Medicine, State Key Laboratory of Radiation Medicine and Protection, Key Laboratory of Stem Cells and Medical Biomaterials of Jiangsu Province, Medical College of Soochow University, Suzhou, 215123 Jiangsu China; 2 CAS Key Laboratory of Tissue Microenvironment and Tumor, Shanghai Institute of Nutrition and Health, Shanghai Institutes for Biological Sciences, University of Chinese Academy of Sciences, Chinese Academy of Sciences, Shanghai, 200031 China; 3 Institutes for Translational Medicine, State Key Laboratory of Radiation Medicine and Protection, Soochow University, Suzhou, 215123 Jiangsu China

**Keywords:** Macrophages, hADSCs, Adipogenesis, Inflammation cytokines

## Abstract

**Background:**

Mesenchymal stem/stromal cells (MSCs) and macrophages are critical components in many tissue microenvironments, including that in adipose tissue. The close interaction between MSCs and macrophages modulates various adipose-related disease development. However, the effects of macrophages on the fate of MSCs remain largely elusive. We here studied the effect of macrophages on the adipogenic differentiation of MSCs.

**Methods:**

Macrophages were obtained from THP-1 cells treated with phorbol-12-myristate-13-acetate (PMA). The induced matured macrophages were then induced to undergo classically activated macrophage (M1) or alternatively activated macrophage (M2) polarization with Iipopolysaccharide (LPS)/interferon (IFN)-γ and interleukin (IL)-4/IL-13, respectively. The supernatants derived from macrophages under different conditions were applied to cultured human adipose tissue-derived mesenchymal stem/stromal cells (hADSCs) undergoing adipogenic differentiation. Adipogenic differentiation was evaluated by examining Oil Red O staining of lipid droplets and the expression of adipogenesis-related genes with real-time quantitative polymerase chain reaction (Q-PCR) and western blot analysis.

**Results:**

The adipogenic differentiation of hADSCs was impaired when treated with macrophage-derived supernatants, especially that from the M1-polarized macrophage (M1-sup). The inhibitory effect was found to be mediated by the inflammatory cytokines, mainly tumor necrosis factor-α (TNF-α) and IL-1β. Blocking TNF-α and IL-1β with neutralizing antibodies partially alleviated the inhibitory effect of M1-sup.

**Conclusion:**

Macrophage-derived supernatants inhibited the adipogenic differentiation of hADSCs in vitro, irrespective of the polarization status (M0, M1 or M2 macrophages). M1-sup was more potent because of the higher expression of pro-inflammatory cytokines. Our findings shed new light on the interaction between hADSCs and macrophages and have implications in our understanding of disrupted adipose tissue homeostasis under inflammation.

## Background

Mesenchymal stem/stromal cells (MSCs), a heterogeneous stem cell population, were first found in bone marrow by Friedenstein et al. [1] and were subsequently isolated from various other tissues, such as adipose tissue, umbilical cord and dental pulp [2, 3]. MSCs are now characterized by their abilities to self-renew and give rise to multiple lineages including osteoblasts, chondrocytes and adipocytes [4–6]. As a major source of adipocytes, MSCs first differentiate into preadipocytes that are committed to the adipogenic lineage. Preadipocytes then give rise to enlarged mature adipocytes that can synthesize lipid droplets, secrete specific adipocyte factors and regulate energy metabolism [7]. Adipocyte differentiation from MSCs is believed to play a vital role in maintaining the adipose tissue homeostasis [8]. Recently, emerging evidence has demonstrated that MSCs interact with both innate and adaptive immune systems to modulate local immune response [9]. IFN-γ in combination with any one of TNF-α/IL-1α/IL-1β can endow MSCs with immunomodulatory capability, mainly in an indoleamine 2, 3-dioxidase (IDO)/inducible nitric oxide synthase (iNOS)-dependent manner hinging on species difference [10, 11]. Accordingly, MSCs-based cell therapy can modulate immune microenvironment and dampen immune and inflammatory responses associated with graft-versus-host-disease (GVHD) [12], systemic lupus erythematosus (SLE) [13] and experimental autoimmune encephalomyelitis (EAE) [14].

Macrophages, a critical component in tissue microenvironment, contribute to the maintenance or disruption of homeostasis via the functionally distinct subpopulations in response to different microenvironmental cues [15]. There are two main types of activation and polarization states in mammals: M1 and M2 [16, 17]. The imbalance between M1 and M2 macrophages has been found to be responsible for chronic inflammatory milieu in adipose tissue and insulin sensitivity [18]. In lean individuals, macrophages dispersed throughout adipose tissues are predominantly resident macrophages and are polarized toward M2 phenotype, while in obese individuals there are an elevated number of infiltrating macrophages of activated pro-inflammatory phenotype, namely M1 subtype, in adipose tissue [19]. Interestingly, macrophages are remarkably plastic, the polarized M1/M2 phenotype can, to some extent, be experimentally reversed in vitro and in vivo, which makes macrophages as effect target for immunomodulatory therapeutic applications [20]. Local cytokines milieu, reactive oxygen species (ROS) and metabolism pathway can all direct macrophage polarization [21, 22].

Adipose tissues are responsible for storing energy and consist of a large number of clusters of fat cells and immune cells, such as macrophages, as mentioned above [23]. Macrophages play an indispensable role in maintaining adipose-tissue homeostasis. Zheng et al. demonstrated that macrophage accumulation in adipose tissue during obesity is initiated by in situ proliferation of resident adipose tissue macrophages (ATMs) and further augmented by monocyte migration and subsequent macrophage differentiation in the late stage [24]. Furthermore, CD11b (integrin α_M_) deficiency resulted in impaired monocyte migration and improved insulin resistance (IR) [25]. However, the interaction between adipocyte precursor, namely MSCs, and macrophages in adipose tissue in situ is still elusive. Recently, the complex cross‐talk between MSCs and macrophages has attracted significant attention. MSCs respond to macrophages and then affect their polarization by secreting factors or other effector [26], such as IL-6 [27], prostaglandin E2 (PGE2) [28] and exosome [29]. Extensive studies have shown that macrophages could also promote the proliferation of MSCs and possessed the capability to enhance osteogenesis of MSCs in an oncostain M (OSM) signaling pathway dependent manner [30], indicating a critical interaction between MSCs and macrophages under the condition of physiological response during bone injury and bone remodeling.

Here, we studied the effect of macrophages on the adipogenic differentiation of human hADSCs in vitro. We observed a reduced adipogenic differentiation in hADSCs treated with macrophage-derived culture supernatants, especially that from M1 macrophage. Furthermore, inflammatory cytokines TNF-α and IL-1β secreted by M1 macrophage were found to account for the inhibitory effect. These findings expand our understanding of cellular interaction between hADSCs and macrophages.

## Results

### Characterization of polarized macrophage

Human THP-1 monocyte cells were treated with PMA to generate macrophages (Additional file 1: Fig. S1a), as previously reported [31]. Mature macrophages were confirmed by flow cytometry to express CD11b upon PMA treatment (M0 macrophage) compared with control THP-1 (Additional file 1: Fig. S1b). M0 macrophages were then polarized toward M1 and M2 with LPS/IFN-γ and IL-4/IL-13, respectively. M1-polarized macrophage exhibited significantly higher expressions of IL-1β, IL-12, IL-6 and TNF-α than the M0 and M2 macrophages (Additional file 1: Fig. S1c–f). In contrast, M2 macrophages were shown to highly express CC chemokine ligand 22 (CCL22), transglutaminase 2 (TGM2) and peroxisome proliferator-activated receptor γ (PPAR-γ) (Additional file 1: Fig. S1g–i).

### Macrophage-derived supernatants inhibit adipocyte differentiation of hADSCs

hADSCs at 3th passage were identified by their expression of MSC surface markers through flow cytometry assay. These cells were positive for CD73, CD105, CD90, CD29 but negative for CD11b, CD31, CD34, CD45 (Additional file 1: Fig. S2a). Furthermore, we cultured hADSCs in differentiation medium and examined their adipogenic and osteogenic potentials by Oil Red O or Alizarin Red S staining, respectively (Additional file 1: Fig. S2b). They could be induced to exhibit enormous lipid droplets and increased calcium accumulation, respectively.

**Fig. 1:**
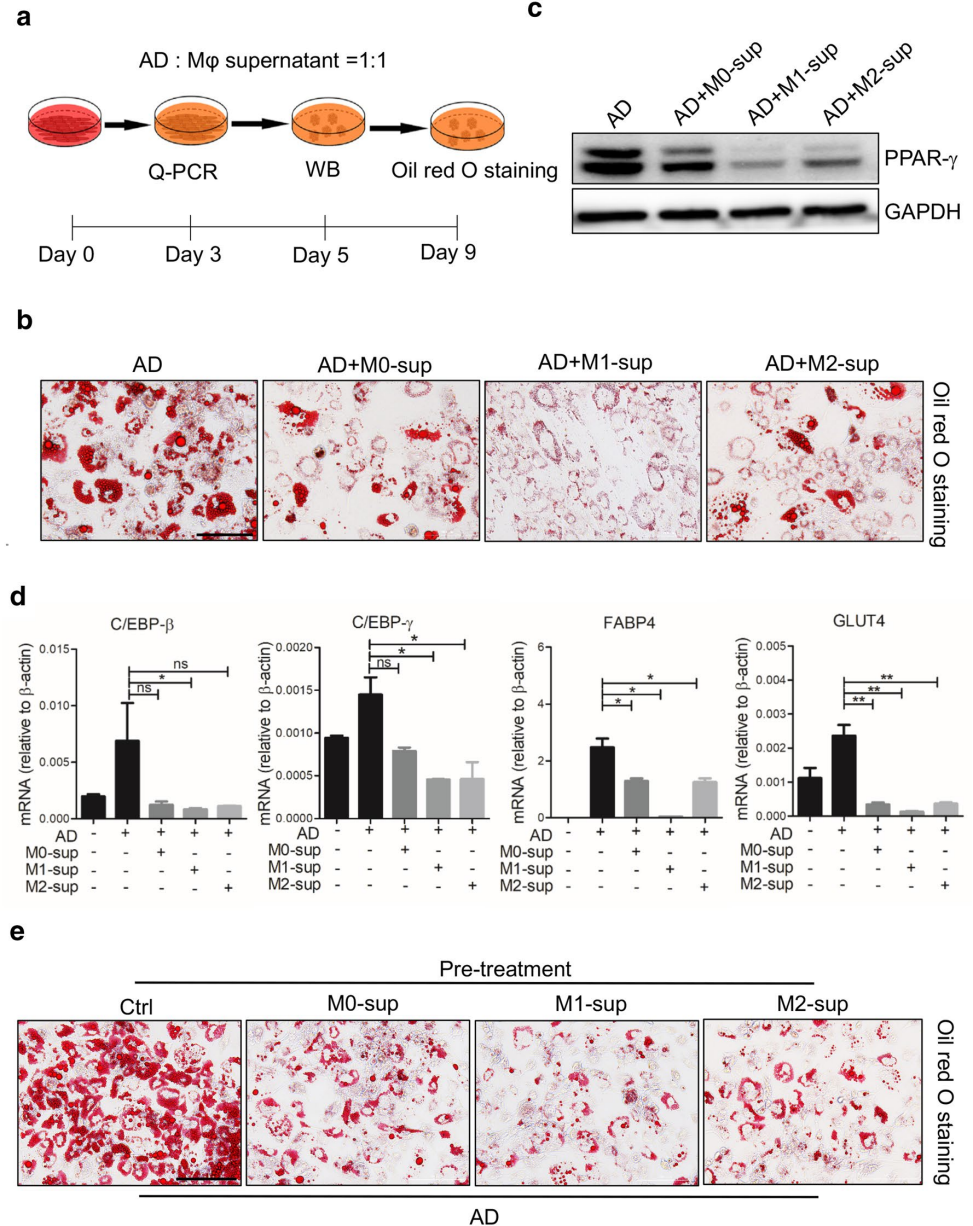
Macrophage-derived supernatants inhibit adipocyte differentiation of hADSCs. **a** Scheme of the experimental procedure. hADSCs (2 × 10^4^) were plated and cultured with adipogenesis-inducing medium (AD) with or without the supernatants from different macrophage subtypes at the ratio of 1:1 in 48-well plate, medium was changed every 3 days. **b** hADSCs cells that were induced to undergo adipogenic differentiation were fixed for triglycerides staining with Oil Red O to show lipid droplets. **c** Western blot analysis of the protein levels of PPAR-γ in hADSCs cultured in different conditions for 5 days. **d** Expression of adipogenic genes were measured by Q-PCR assay on day 3. **e** hADSCs were pre-treated with the specific supernatant for 24 h, then changed to adipogenic inducing medium without supernatant, lipid droplets in hADSCs were revealed by Oil Red O staining after 9 days. Scale bars are 100 μm

To ascertain the effect of macrophages on adipogenic differentiation of hADSCs in vitro, M1 and M2 macrophages were prepared and fresh culture supernatants were respectively collected 24 h after removal of stimuli. The adipogenic differentiation of hADSCs was evaluated in the presence of different macrophage supernatants (M0-sup, M1-sup or M2-sup) (Fig. 1a). After 9-day differentiation, a remarkably repressed formation of lipid droplets was observed in the hADSCs treated with macrophage supernatants, especially M1-sup (Fig. 1b). Consistently, a dramatic decrease in the expression of adipogenic differentiation related genes was observed, such as PPAR-γ, glucose transporter 4 (Glut4), CCAAT/enhancer binding proteins β (C/EBP-β), CCAAT/enhancer binding proteins γ (C/EBP-γ) (Fig. 1c, d). Interestingly, we found that pre-treatment of macrophage-conditioned supernatants also endowed hADSCs resistance to adipogenic differentiation (Fig. 1e), indicating that the inhibitory effect did not require the continuous presence of the supernatant. Taken together, these results suggested that the macrophage supernatants, irrespective of the polarization status of the macrophages, impaired the adipogenic differentiation potential of hADSCs.

### The inhibitory effect of macrophage culture supernatants is dependent on secretory proteins

**Fig. 2:**
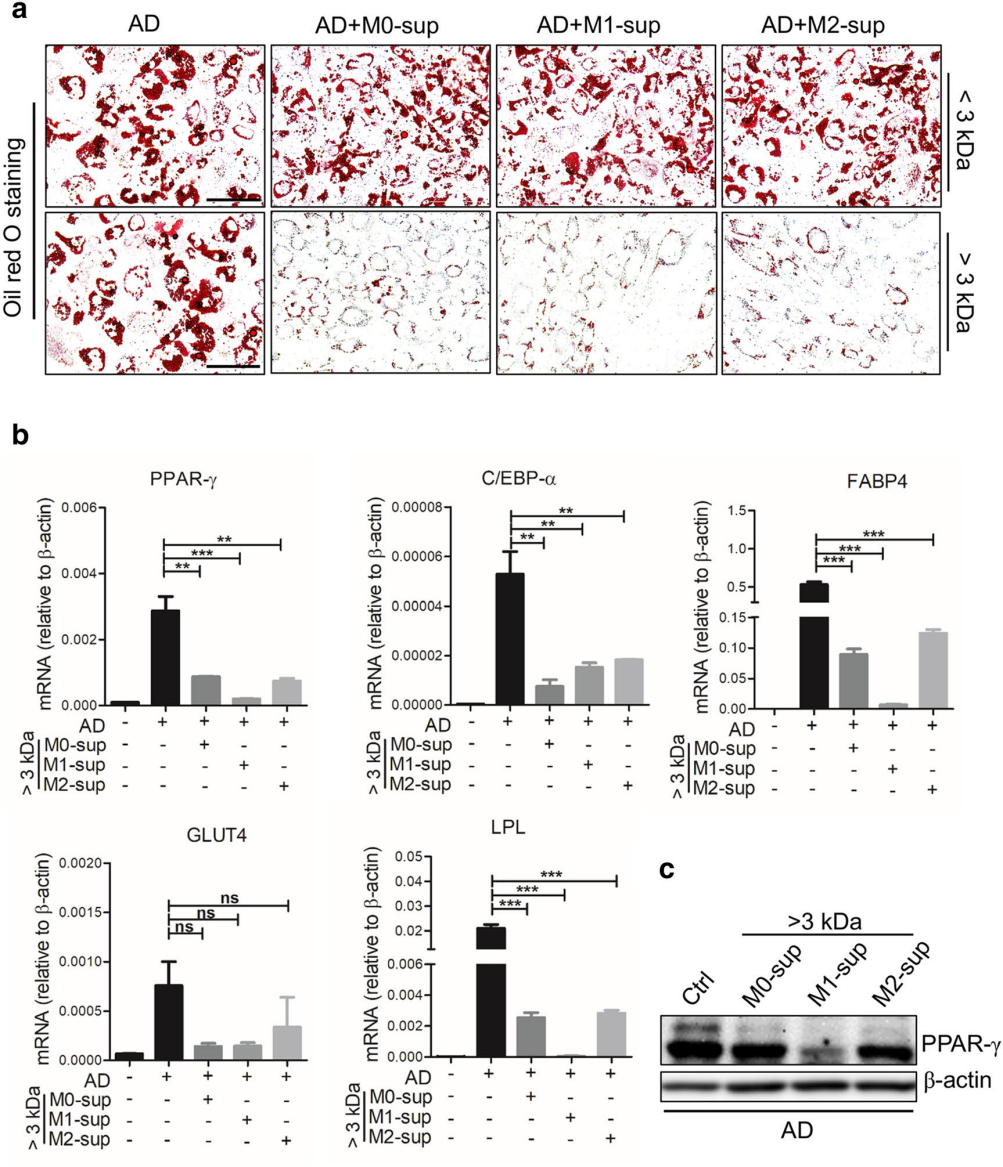
The adipogenesis-inhibiting effect of macrophage supernatants is dependent on secretory macromolecule. **a** hADSCs were cultured with adipogenesis-inducing medium (AD) with or without the supernatant from different macrophage subtypes (> 3 kDa or < 3 kDa) at the ratio of 1:1 in 48 plate, medium was changed every 3 days. After being cultured for 9 days, cells were fixed for triglycerides staining with Oil Red O to show lipid droplets. **b** Expression of adipogenesis-related genes in hADSCs treated with macrophages-derived supernatant (> 3 kDa) was measured by Q-PCR on day 3. **c** Western blot analysis of the protein levels of PPAR-γ in hADSCs cultured with different condition medium. All scale bars, 100 μm

The components in macrophage supernatants may include cytokines, neutral proteases, chemokines, complement components, arachidonic acid metabolites and lactic acid. To further determine the components which are responsible for the inhibitory effect on adipogenesis of hADSCs, ingredients were divided into two fractions, one with components > 3 kDa and one with < 3 kDa. We found that only the ingredients in the > 3 kDa, but not those in the < 3 kDa, could inhibit the formation of lipid droplets, as shown by Oil Red O staining (Fig. 2a). Accordingly, we found decreased expression of PPAR-γ, C/EBP-α and C/EBP-β in hADSCs treated with > 3 kDa fraction (Fig. 2b, c). In addition, the M1-sup was more potent than M0/M2-sup in inhibiting adipocyte differentiation. These data indicate that large molecules, rather than small molecules or metabolites, produced by macrophages were responsible for the inhibitory effect on adipogenic differentiation of hADSCs.

### Pro-inflammatory cytokines TNF-α and IL-1β inhibit adipocyte differentiation of hADSCs

**Fig. 3:**
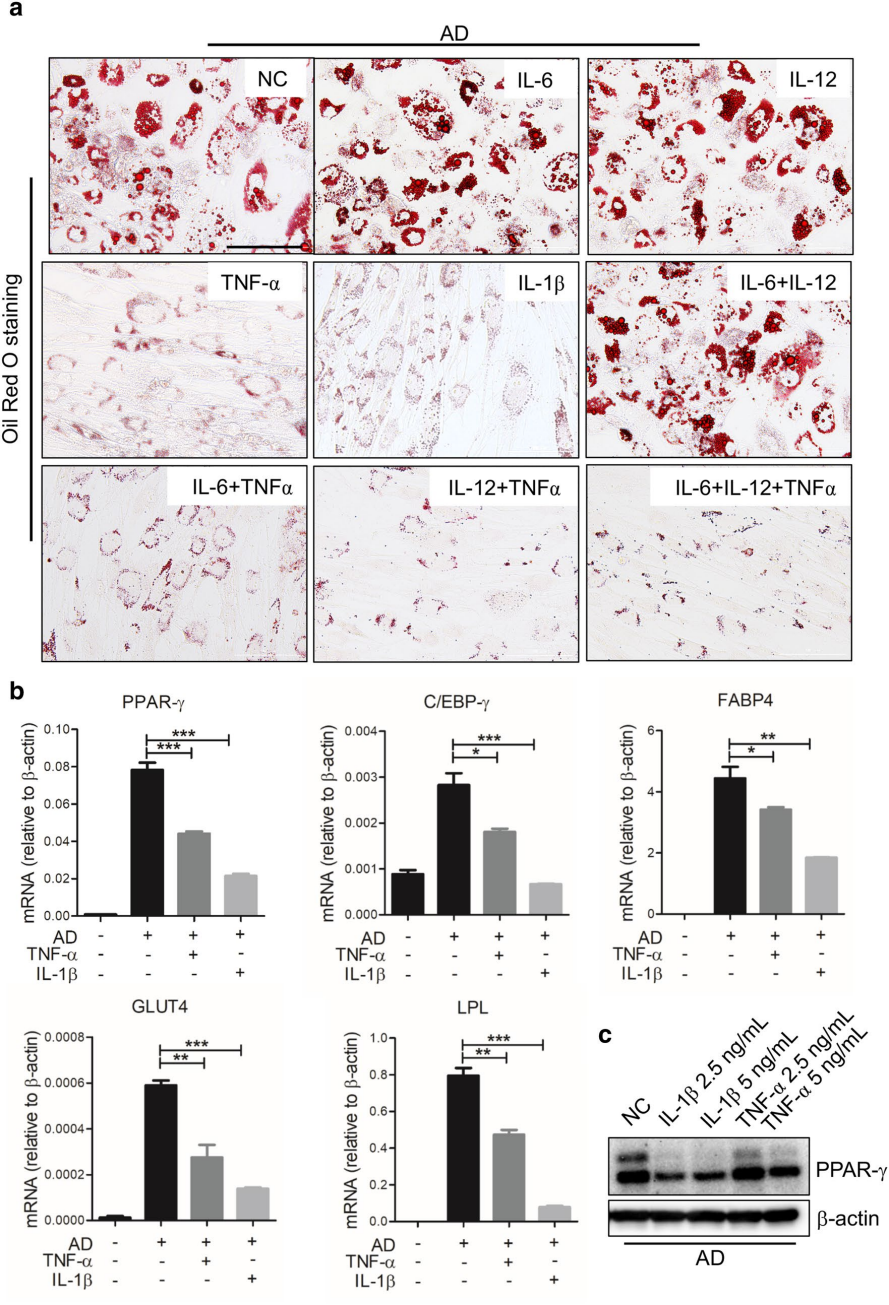
TNF-α and IL-1β repress the adipogenesis of hADSCs. **a** hADSCs were cultured with adipogenesis-inducing medium (AD) with or without IL-6, IL-12, TNF-α and IL-1β (5 ng/mL each), respectively. Medium was changed every 3 days. After being cultured for 9 days, cells were fixed for triglycerides staining with Oil Red O. **b** Expression of adipogenesis-related genes in hADSCs treated with TNF-α/IL-1β (0.1 ng/mL each) was determined by Q-PCR assay on day 3. **c** Western blot analysis of PPAR-γ in adipogenic hADSCs interfered with TNF-α or IL-1β cytokines at different concentration. Scale bars, 100 μm

The accumulation of adipose tissue macrophages (ATMs) is a significant characteristic of obesity-associated chronic inflammation, the ATMs are also critical in regulating obesity development. Macrophages form the so-called ‘crown-like structures’(CLSs) around adipocytes during obesity, and mainly exhibit M1 phenotype [19, 32]. Considering that the M1 macrophages produce various types of inflammatory cytokines, such as TNF-α and IL-1β, which play an important role in the propagation of obesity-related inflammation [33], we thus focused on the pro-inflammatory cytokines in M1-sup. TNF-α, IL-6, IL-12 and IL-1β, alone or in combination, were added to hADSCs during the induction of adipogenic differentiation. We found that the adipocyte differentiation of hADSCs was significantly inhibited by TNF-α or IL-1β treatment in a concentration-dependent manner, as shown by Oil Red O staining and Q-PCR assay (Fig. 3a, Additional file 1: Fig. S3a–c). However, the adipogenic differentiation of hADSCs was not altered by IL-6 and IL-12, when they were used alone or in combination (Fig. 3a). Next, Q-PCR assay showed that key adipogenesis related genes, including PPAR-γ, C/EBP-α, Glut4 and lipoprotein lipase (LPL), were also evidently decreased by TNF-α or IL-1β at a very lower concentration during adipogenic differentiation (Fig. 3b). Furthermore, Western blot assay further confirmed that TNF-α and IL-1β inhibited the expression of PPAR-γ, a master transcription factor for adipogenesis, in differentiated hADSCs (Fig. 3c). Thus, TNF-α and IL-1β, cytokines that are highly expressed in M1 polarized macrophage, can potently inhibit the adipocyte differentiation of hADSCs.

### TNF-α and IL-1β mediate the anti-adipogenic effect of M1-sup

**Fig. 4:**
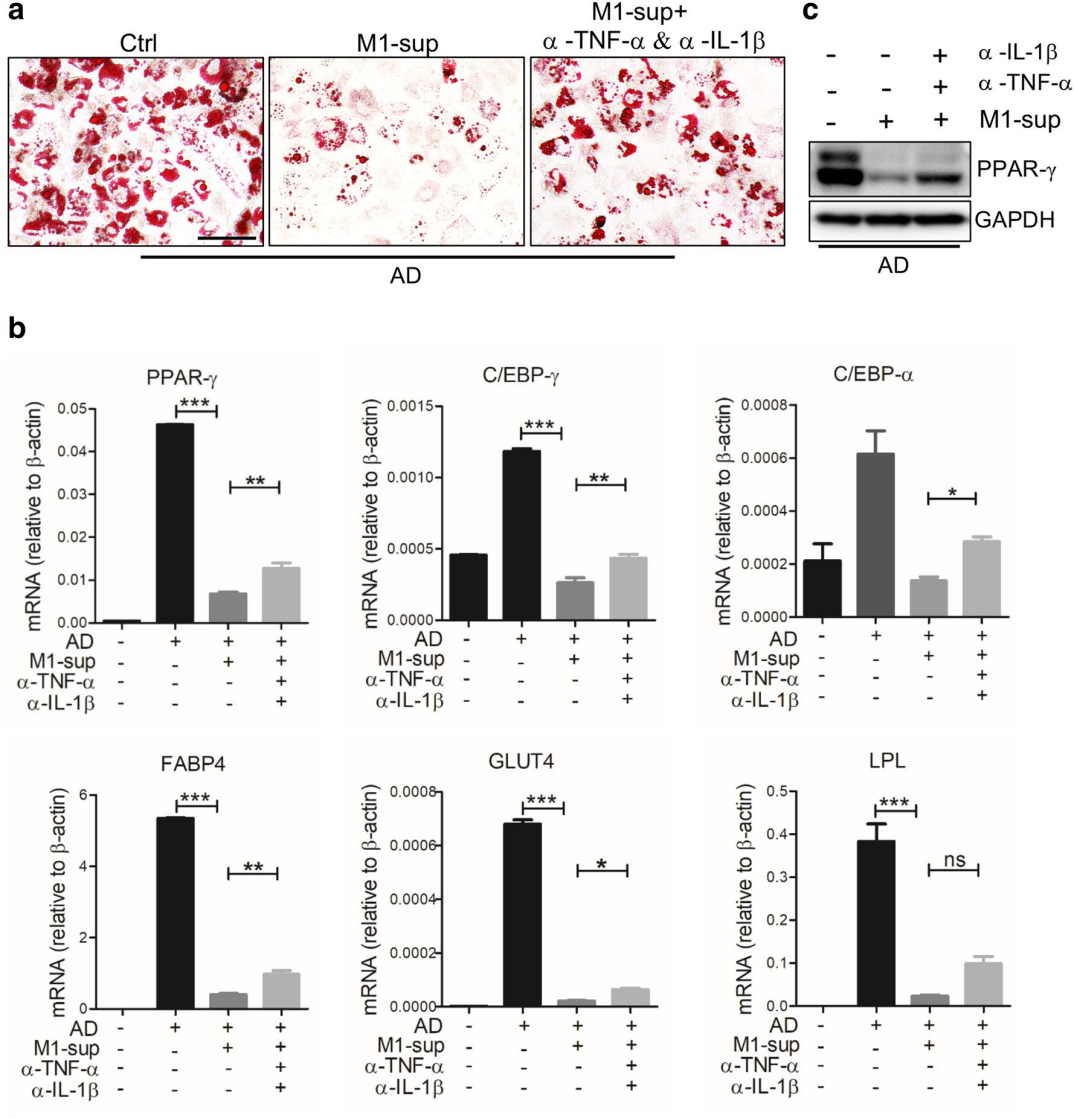
Macrophages restrict adipogenic differentiation of hADSCs through TNF-α and IL-1β production. **a** Antibody mixture (Abs) containing anti-TNF-α and anti-IL-1β (2 μg/mL each) partially relieves the inhibition of adipogenic differentiation of hADSCs by M1-sup. Cells were stained with Oil Red O after being cultured for 9 days. **b** Q-PCR was used to assess the expression of PPAR-γ, C/EBP-γ, C/EBP-α, FABP4, Glut4, LPL in hADSCs. **c** Western blot analysis of PPAR-γ in adipogenic hADSCs treated with TNF-α and IL-1β neutralizing antibodies in combination. Scale bars, 100 μm

Given that pro-inflammatory cytokines TNF-α and IL-1β can inhibit adipogenic differentiation of hADSCs, we next investigated whether the two cytokines were responsible for the adipogenesis-inhibiting effect of M1-sup. We induced adipogenic differentiation in hADSCs in the presence of M1-sup as described above, but with specific antibodies targeting TNF-α and IL-1β being added. We found that neutralization of these cytokines abolished the anti-adipogenic effect of M1-sup, as significantly more lipid droplets were observed when compared with hADSCs treated only with the M1-sup (Fig. 4a). The repression of lipogenic genes, such as PPAR-γ, Glut4, FABP4 and C/EBP-α, by M1-sup was also partially reversed with anti-TNF-α and anti-IL-1β blockage (Fig. 4b, c). It is worth noting that the failure to fully abrogate the inhibition of M1-sup may result from an incomplete blockade of those cytokines. Collectively, data above indicate that pro-inflammatory cytokines TNF-α/IL-1β were responsible for the inhibitory effect of M1-sup. Considering that there are other components in macrophage supernatant, we performed other experiments and found that exosomes secreted by M1 macrophage can also inhibit adipogenic differentiation of hADSCs, which will be reported in a separate study.

## Discussion

Macrophages are now recognized as crucial regulators of physiological and pathological remodeling of adipose tissue. They can clear cellular debris and participate in tissue immune surveillance. Adipose tissue macrophages (ATMs) also function in lipid buffering [34]. Studies have shown that induction of brown adipogenesis with β3-adrenoceptor (ADRB3) agonist treatment is triggered by the recruitment of macrophages to vulnerable white adipocytes undergoing agonist-mediated cell death [35]. Recent work indicates that ATMs can clear adipose of dying adipocytes via arachidonate 15-lipoxygenase [36]. While macrophages play a critical role in regulating the adipose tissue homeostasis, how macrophages regulate adipogenesis remains to be fully addressed.

**Fig. 5:**
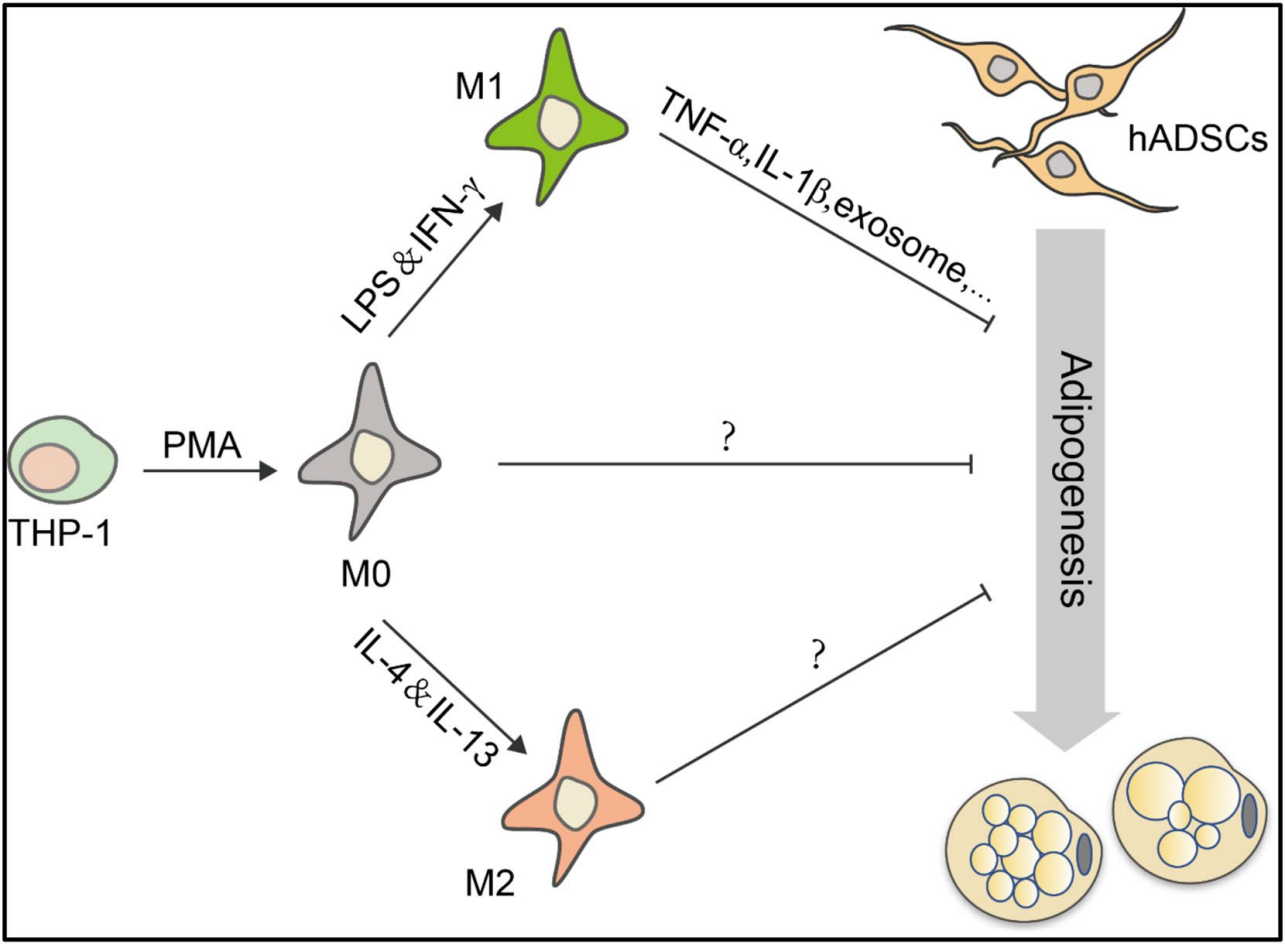
A schematic diagram depicting the inhibitory effects of macrophages on adipocyte differentiation of hADSCs. M0, M1 and M2 macrophages can all inhibit adipogenic differentiation of hADSCs. M1 macrophage exert inhibitory effects by secreting inflammatory cytokines TNF-α and IL-1β

We tested the effects of M0, M1 or M2-polarized macrophages derived from THP1 monocytes on the lipogenic behavior of hADSCs in vitro. The results showed that they can all inhibit adipogenic differentiation, although the M1 macrophages are most potent. M1 macrophages were found to exert their inhibitory effect through secreting TNF-α and IL-1β, but not IL-12 and IL-6. These in vitro results are in contrast to a report from Zhu et al. who demonstrated that ADSCs from obese pigs showed enhanced adipogenic propensity, which was abolished by anti-TNF-α antibody treatment, additionally, ADSCs from lean pigs showed enhanced adipogenesis when treated with TNF-α [37]. This difference may result from species difference or different ADSCs status originating in obese or lean individual. However, our finding is consistent with the report that TNF-α inhibits adpogenic differentiation of ADSCs [38]. It appears that how macrophages act on ADSCs under various physiological and pathological conditions still needs to be further explored.

Exosomes, a type of extracellular vesicles, play an important role in cellular communication as they are rich in mRNA, miRNA, and proteins that can regulate cellular signaling [39, 40]. Previous studies have report that exosomes secreted by adipose-tissue specific macrophages in obese patients regulate the phosphorylation of AKT signaling pathway by inhibiting the expression of PPAR-γ and Glut4, thus affect the insulin sensitivity of the body [41]. It is possible that exosomes derived from macrophages may also have an impact on adipogenic differentiation of hADSCs.

While we demonstrated an inhibitory effect of macrophages on the adipocyte differentiation of hADSC in vitro, whether the findings can be translated to the physiological and pathological conditions in vivo remains to be verified. Mouse models with the cytokine receptors being specifically deleted in ADSCs may help to confirm the role of inflammatory cytokines during adipogenesis. How different subsets of macrophages interact with ADSCs in vivo also needs to be delineated. Does the inhibition of adipocyte differentiation by cytokines have a beneficial effect? Is it possible that the suppression of adipocyte differentiation would endow the ADSCs an enhanced function in maintaining tissue homeostasis? It is well documented that MSCs exposed to inflammatory cytokines can acquire an increased immunomodulatory function [9]. A better understanding of the interaction between macrophages and hADSCs may help the clinical management of obesity and related diseases.

Taken together, we demonstrated that pro-inflammation cytokines derived from M1 macrophage inhibit the adipogenic differentiation of hADSCs (Fig. 5). Given that TNF-α/IL-1β can also endow MSCs immunosuppressive function, we speculate that there might be a counterbalance between adipogenesis potential and immunosuppressive function of MSCs, namely, the anti-adipogenic effect of pro-inflammatory cytokines may be evolutionary embedded to facilitate the immunosuppressive function of MSCs.

## Conclusion

Our study demonstrated an inhibitory effect of macrophages on the adipogenic differentiation of hADSCs in vitro. While M0, M1 and M2 polarized macrophages can all inhibit adipocyte differentiation, M1 macrophages possess the most potent effect that is mediated by TNF-α and IL-1β. Our findings expand the knowledge about the interaction between macrophages and hADSCs as well as the fate determination of hADSCs.

## Materials and methods

### Cell line

THP-1 cells, which were derived from the peripheral blood of a one-year-old boy with acute monocytic leukemia (American Type Culture Collection, ATCC), were cultured with RPMI-1640 medium (Hyclone) supplemented with 10% fetal bovine serum (FBS, Hyclone), 100 U/mL penicillin and 100 g/mL streptomycin (Invitrogen) in a humidified incubator under 5% CO_2_ at 37 °C.

### Induction of THP-1 derived macrophages

The macrophages were obtained by treating THP-1 cells with PMA (Sigma, USA) (100 ng/mL) for 48 h. The matured macrophages were exposed to LPS (100 ng/mL) and IFN-γ (20 ng/mL) for 48 h to obtain M1 polarized macrophage (classical activated macrophage). M2-polarized macrophage (alternatively activated macrophage) were obtained by treatment with IL-4 and IL-13 (20 ng/mL each) for 48 h. All of the cytokines were from PeproTech, USA.

### Isolation and culture of hADSCs

ADSCs were obtained from the adipose tissue of lipoaspirate samples following the protocols approved by the Ethics Committee of Soochow University. Informed consent was obtained from each patient. Fresh adipose tissues were washed with phosphate-buffered saline (PBS) in a few more times and then digested with collagenase II (Gibco, USA) for 60–90 min at 37 °C in a shaker. After centrifuging at 500 g for 8 min, the stromal vascular cell fraction suspension was cultured with DME/F12 medium (Hyclone, USA) containing 10% FBS, 100 U/mL penicillin, 100 g/mL streptomycin, 2 mM glutamine and basic fibroblastic growth factor (bFGF) (complete medium) in specific T75 flask. The medium was changed every 3 days and cells were routinely passaged by trypsin digestion. hADSCs were characterized for surface markers by flow cytometer at 3th passage.

### Preparation of macrophage supernatants

M0, M1 or M2 macrophages were obtained as described above. Cells were washed with PBS three times and cultured with fresh DMEM/High complete medium at 37 °C, 5% CO_2_. Collected the cell culture medium after 2 days and centrifuged at 2000 × *g*, 4 °C for 10 min. Discarded precipitates and supernatants were then transferred to molecular weight cut-off filter basing 3 kDa and centrifuged at 5000 × *g*, 4 °C for 30 min. Finally, fractions greater than 3 kDa (the upper layer) and less than 3 kDa (the bottom layer) were separately collected and stored at − 80 °C.

### Adipogenic and osteogenic differentiation of hADSCs

For adipogenic differentiation, hADSCs (2 × 10^4^/well) were seeded into 48-well plate with DME/F12 complete medium. When cells were grown to 80% confluence, medium was changed to adipogenic medium consisting of 0.5 mM 3-isobutyl-1-methylxanthin (IBMX, Sigma, USA), and 60 μM indomethacin (Sigma, USA), 100 nM dexamethasone (Sigma, USA), 10 μg/mL insulin (Sigma, USA). Then adipogenic medium was changed every 3 days. Osteogenic differentiation of hADSCs was induced according to established protocols with minor modification. hADSCs were cultured with DMEM/low medium containing 10 nM dexamethasone, 100 μM L-ascorbic acids (Sigma, USA), 10 nM β-glycerophosphate (Sigma, USA), the medium was changed every 3 days during the osteogenesis-inducing process.

### Oil Red O staining

hADSCs that were induced to undergo adiogenic differentiation were fixed on day 9 with 4% paraformaldehyde for 15–30 min at room temperature and washed with PBS twice, then cells were stained with Oil Red O (Bio-Connect, Holland) working solution for 15 min and washed by double distilled water for at least 3 times.

### RNA isolation and real-time quantitative polymerase chain reaction (Q-PCR)

Total RNAs were extracted from cells by Trizol reagent (Thermo, USA) according to the manufacturer’s instructions. About 1μg of total RNAs from each sample were reverse-transcribed into cDNA using the PrimeScript™ RT reagent Kit (TaKaRa, Japan). The mRNA expressions were quantitatively assessed by SYBR Green-based Q-PCR in an Applied Biosystems PRISM 7900HT Fast Real-time PCR System. The primers were synthesized by Suzhou GENEWIZ institute. RNA expression levels were compared after normalization with β-actin. The primer sequences were shown in Table 1.

**Table 1:** List of primers used for quantitative polymerase chain reaction (β-actin was used as an internal control)

Gene	Primer sequences
PPAR-γ	
Forward	5′-TACTGTCGGTTTCAGAAATGCC-3′
Reverse	5′-GTCAGCGGACTCTGGATTCAG-3′
C/EBP-α	
Forward	5′-CGAAGAGACGGCCCTTGCTG-3′
Reverse	5′-GGGATACATCCTCAGGGCCACA-3′
C/EBP-β	
Forward	5′-CTTCAGCCCGTACCTGGAG-3′
Reverse	5′-GGAGAGGAAGTCGTGGTGC-3′
C/EBP-γ	
Forward	5′-ACTCCAGGGGTGAACGGAAT-3′
Reverse	5′-CATGGGCGAACTCTTTTTGCT-3′
Glut4	
Forward	5′-TGGGCGGCATGATTTCCTC-3′
Reverse	5′-GCCAGGACATTGTTGACCAG-3′
LPL	
Forward	5′-TCATTCCCGGAGTAGCAGAGT-3′
Reverse	5′-GGCCACAAGTTTTGGCACC-3′
FABP4	
Forward	5′-ACTGGGCCAGGAATTTGACG-3′
Reverse	5′-CTCGTGGAAGTGACGCCTT-3′
IL-6	
Forward	5′-CAGCCCTGAGAAAGGAGACATG-3′
Reverse	5′-GGTTGTTTTCTGCCAGTGCCT-3′
IL-12	
Forward	5′-GATGGCCCTGTGCCTTAGTA-3′
Reverse	5′-TCAAGGGAGGATTTTTGTGG-3′
IL-1β	
Forward	5′-GACCTGAGCACCTTCTTTCCCT-3′
Reverse	5′-CATCGTGCACATAAGCCTCGT-3′
TNF-α	
Forward	5′-GACAAGCCTGTAGCCCATGTTG-3′
Reverse	5′-TGGTTATCTCTCAGCTCCACGC-3′
TGM2	
Forward	5′-CAAGGCCCGTTTTCCACTAAG-3′
Reverse	5′-GAGGCGATACAGGCCGATG-3′
CCL22	
Forward	5′-ATCGCCTACAGACTGCACTC-3′
Reverse	5′-GACGGTAACGGACGTAATCAC-3′
β-actin	
Forward	5′-TTGCCGACAGGATGCAGAAGGA-3′
Reverse	5′-AGGTGGACAGCGAGGCCAGGAT-3′

### Western blot analysis

The poly vinylidene fluoride (PVDF) membrane was blocked with 2% bovine serum albumin (BSA) solution or 5% nonfat dried milk and then incubated with primary antibodies overnight at 4 °C. Membrane was washed with 1 × tris-buffered saline and tween 20 (TBST) and incubated with either anti-rabbit or anti-mouse IgG horseradish peroxidase-conjugated secondary antibody and finally exposed using ChemiDoc XRS imaging system. Western blot data shown in figures were representative of more than three independent experiments.

### Flow cytometry analysis

Cells were harvested by trypsin digestion and washed with PBS twice and then stained with fluorescein isothiocyanate-conjugated or phycoerythrin-conjugated (monoclonal) antibodies for 30 min at 4 °C, after incubation, cells were washed twice and resuspended in 200 μL PBS. Finally, cells were detected by flow cytometer (BECKMAN, USA). Data was analyzed using Flowjo software.

### Quantification and statistical analysis

Results were expressed as mean ± SEM. The difference between various treatments were evaluated by either the two tailed Student^’^s test or one-way ANOVA with Bonferroni post-test. Data analyses were performed using Graph Pad Prism software version 5.0. A value of *p *< 0.05 was considered statistically significant.

## Supplementary information

**Additional file 1.** Additional figures.

## Data Availability

The datasets used and/or analyzed during the current study are available from the corresponding author on reasonable request

## Abbreviations

hADSCs: Human adipose derived mesenchymal stem/stromal cells
TNF-α: Tumor necrosis factor α
IFN-γ: Interferon-γ
C/EBP: CCAAT/enhancer-binding protein CCAAT
PPAR-γ: Peroxisome proliferator activated receptor γ
FABP-4: Fatty acid binding protein-4
GLUT4: Glucose transporter 4
LPL: Lipoprotein lipase
PMA: Phorbol-12-myristate-13-acetate
IL: Interleukin
TGM2: Transglutaminase 2
CCL22: CC chemokine ligand 22
