# Supplementary material for: Macrophages inhibit adipogenic differentiation of adipose tissue derived mesenchymal stem/stromal cells by producing pro-inflammatory cytokines
Source: Cell Biosci. 2020 Jul 20;10:88. doi: 10.1186/s13578-020-00450-y (PMC7372775; doi:10.1186/s13578-020-00450-y)

**Figure S1*.*** **Characterization of polarized macrophages.**

(a) THP-1 cells were cultured with growth medium containing PMA (100 ng/mL) for 48 h, cells morphology was observed under microscope and then (b) cell surface marker CD11b was detected by flow cytometry. (c-f) Pro-inflammatory genes IL-1β, IL-12, IL-6, TNF-α in M1 macrophages treated with LPS (100 ng/mL) & IFNγ (20 ng/mL) and (g-i) anti-inflammatory genes CCL22, TGM2, PPAR-γ in M2 macrophages primed by IL-4 & IL-13 (20 ng/mL each) for 48 h were assessed by Q-PCR. Scale bars are 200 μm (the upper) and 100 μm (the bottom).

**Figure S2. The identification of hADSCs.** (a) Classical surface markers of hADSCs were detected by ﬂow cytometry. (b) hADSCs were cultured respectively with adipogenic inducing medium (AD) or osteogenic inducing medium, Oil Red O staining were performed after differentiation for 9 days and Alizarin Red staining were performed after inducing for 21 days, scale bars, 100 μm.

**Figure S3. TNF****-α and IL-1β inhibit the adipogenic differentiation of hADSCs in a certain concentration dependent manner.**

1. hADSCs were cultured with adipogenic inducing medium (AD) which respectively

added with TNF-α or (b) IL-1β, Oil Red O staining were performed after differentiation for 9 days. (c) Adipogenesis-related genes, PPAR-γ, C/EBP-γ, FABP4, Glut4, LPL in hADSCs were measured by Q-PCR.


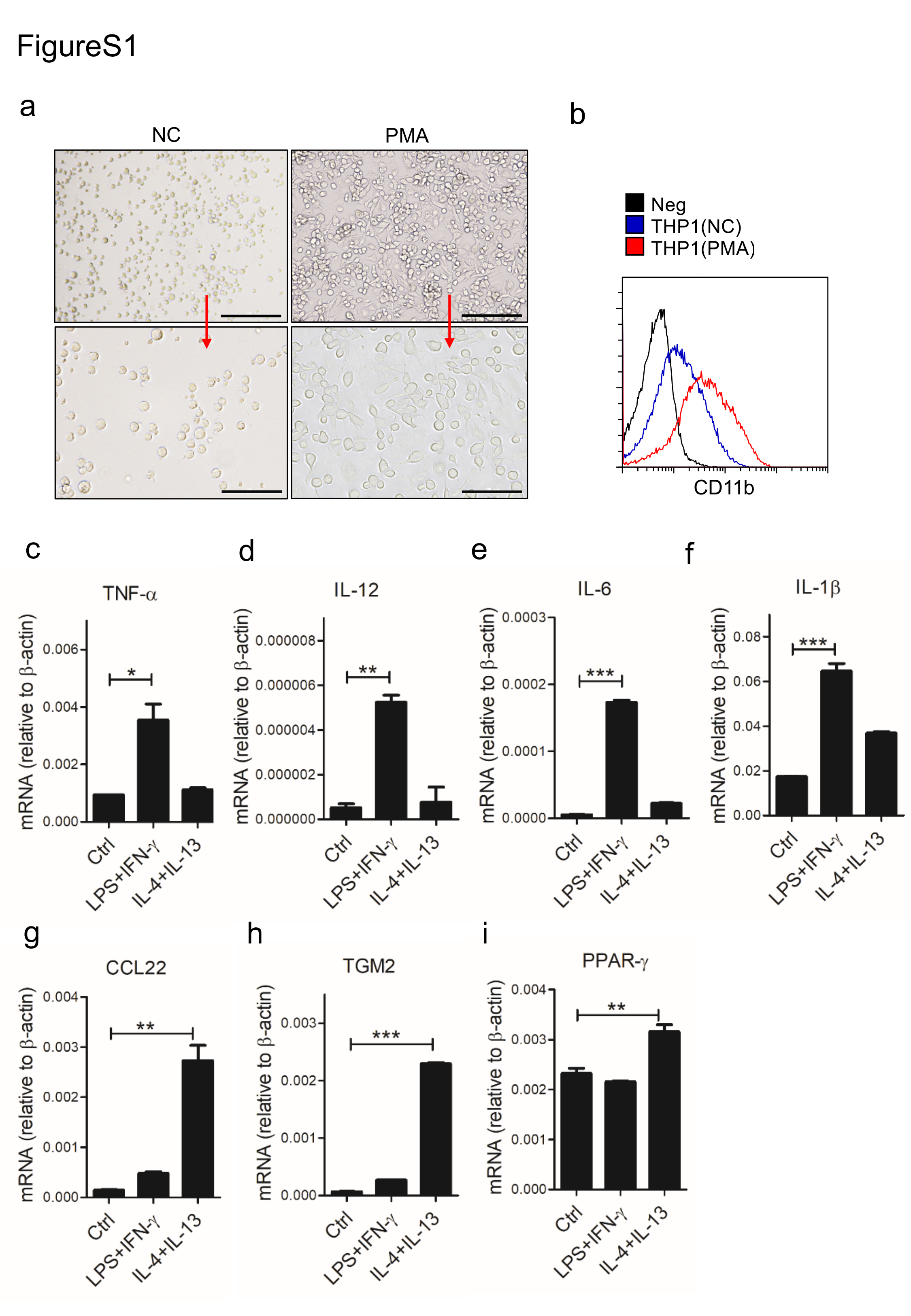

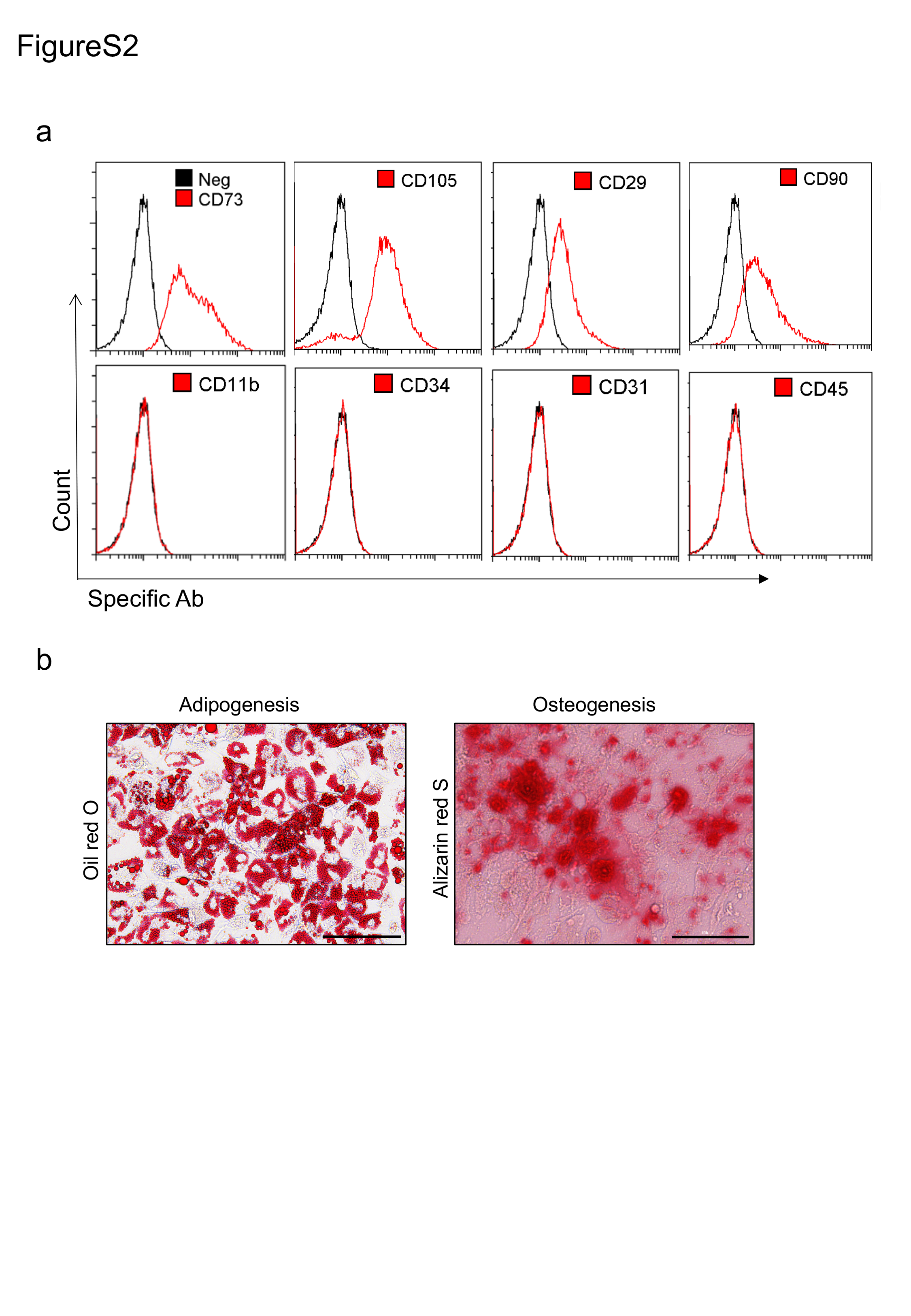

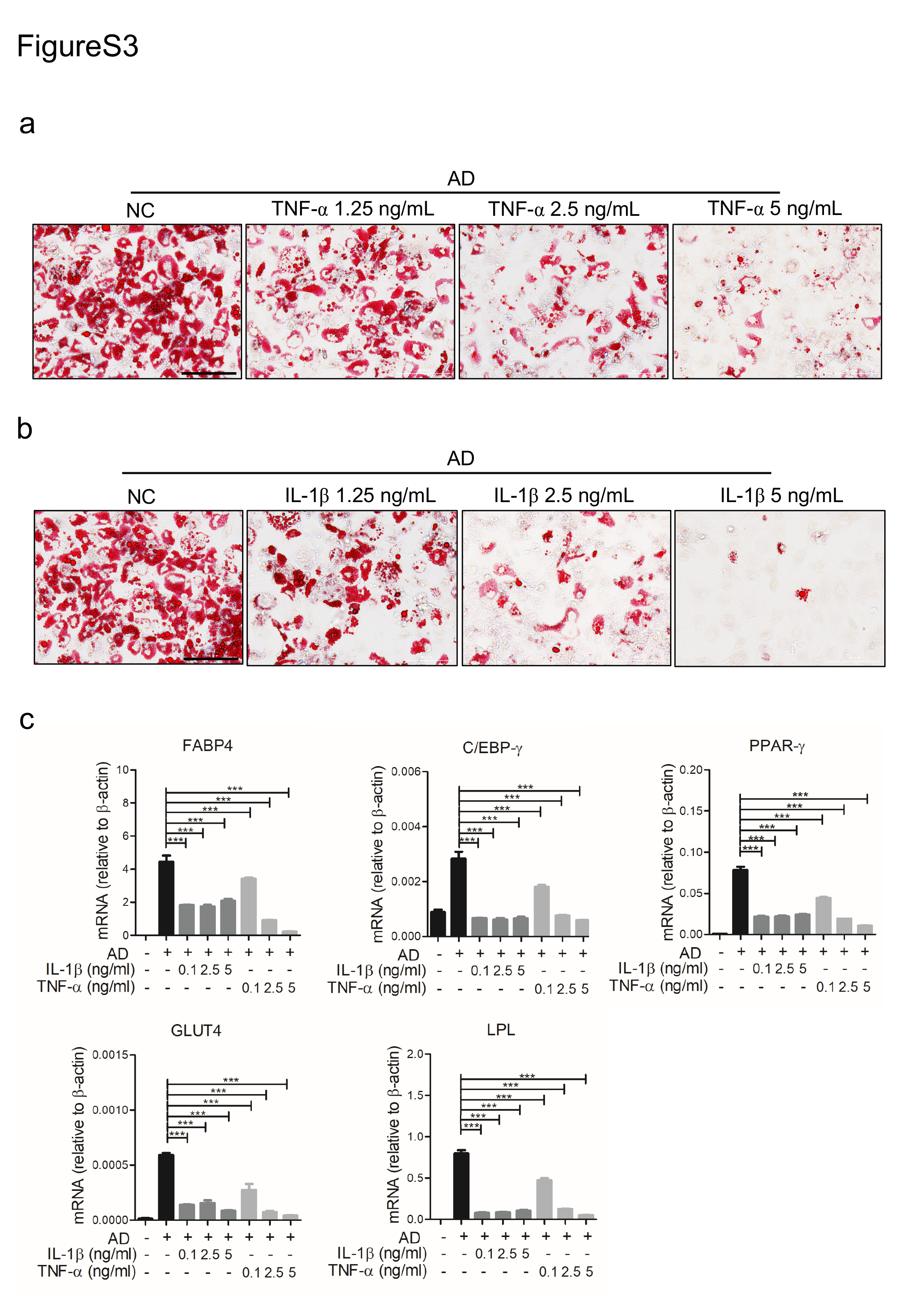

Supplement: Supplementary file 1 — Additional file 1. Additional figures. [file 13578_2020_450_MOESM1_ESM.docx]
